# Effects of a health educational intervention on malaria knowledge, motivation, and behavioural skills: a randomized controlled trial

**DOI:** 10.1186/s12936-019-2676-3

**Published:** 2019-02-20

**Authors:** Ahmed Dahiru Balami, Salmiah Md Said, Nor Afiah Mohd Zulkefli, Norsa’adah Bachok, Bala Audu

**Affiliations:** 1 Department of Community Health, Faculty of Medicine and Health Sciences, Universiti Putra Malaysia, Seri Kembangan, Malaysia; 2 Unit of Biostatistics and Research Methodology, School of Medical Sciences, Universiti Sains Malaysia, Kubang Kerian, Malaysia; 3 Department of Obstetrics and Gynaecology, University of Maiduguri, Maiduguri, Nigeria

**Keywords:** Randomized controlled trial, Health education, Pregnant women, Knowledge, Motivation, Behavioral skills

## Abstract

**Background:**

The levels of insecticide-treated net use among pregnant women and uptake of intermittent preventive treatment in pregnancy, have been sub-optimal in Nigeria. Previous studies have reported positive correlations between knowledge, attitude and practice of malaria preventive measures. It has also been reported that information and motivation, act through a mediator (behavioural skills), to cause a health behaviour change. The aim of this study was as such to develop, implement, and assess the effects of a health educational intervention based on the information–motivation–behavioural skills (IMB) model on the levels of knowledge, motivation, and behavioural skills for ITN use and IPTp uptake among pregnant women in a hospital in north-eastern Nigeria.

**Methods:**

This was a randomized controlled parallel-group trial in which 372 antenatal care attendees were randomly assigned to either an intervention or control group after collecting baseline data using a structured questionnaire. The intervention group received a 4-h health education on malaria, guided by a module developed based on the IMB theory, while the control group received health education on breastfeeding for a similar duration and by the same facilitator. Follow-up data were subsequently collected at 2 months and at 4 months post-intervention using the same questionnaire. The generalized linear mixed models analysis was used to determine the between-group and within-group effects of the intervention. The intention-to-treat analysis was used after missing data had been replaced. This was followed by a sensitivity analysis, where the analyses were repeated without replacing the missing values.

**Results:**

The intervention was significant in achieving a 12.75% (*p *< 0.001), 8.55% (*p *< 0.001), and 6.350% (*p *< 0.001) higher total knowledge, motivation, and behavioural skills scores respectively, for the intervention group over the control group. The sensitivity analysis revealed no great differences in the effect sizes, even when missing data were not replaced.

**Conclusion:**

The intervention module was effective in improving knowledge, motivation and behavioural skills. It is as such recommended to be adopted and incorporated into the routine antenatal health education schedules. It is also recommended that booster doses of the module be given say 2 months after the first dose to sustain levels of motivation and behavioural skills.

*Trial registration* Pan African Clinical Trial Registry, PACTR201610001823405. Registered 26 October 2016, http://www.pactr.org

**Electronic supplementary material:**

The online version of this article (10.1186/s12936-019-2676-3) contains supplementary material, which is available to authorized users.

## Background

Malaria infection during pregnancy is associated with complications like anaemia [1–3], abortion [4, 5], stillbirth [6, 7] low birth weight [8, 9] and pre-term delivery [1]. An estimated 25 million pregnancies are believed to occur annually, in malaria-endemic regions of sub-Saharan Africa [10]. A higher risk of contracting malaria has also been reported among pregnant women [11, 12]. Sleeping under an insecticide-treated net (ITN) every night and receiving at least two doses of intermittent preventive treatment with sulfadoxine-pyrimethamine during pregnancy (IPTp) have been proven to decrease the risk of malaria infection and its complications during pregnancy [13–16] and have been recommended by the World Health Organization (WHO), for all pregnant women in sub-Saharan Africa [10, 17]. However, the level of compliance with these recommended practices has been very poor, as only 18% of pregnant women in Nigeria were reported to sleep under any type of net, while only 23% of women who delivered within the last 2 years preceding the survey had received IPTp [18–25], many of which showed positive associations between these variables [21, 23, 26–29]. Some items of knowledge, motivation and self-efficacy for ITN use, had also been reported to predict ITN use itself [30], suggesting that improvements in these constructs is likely to improve compliance with ITN and IPTp.

A few health educational interventions have been studied among different groups, to determine their effectiveness in improving malaria knowledge and preventive practices among the target groups [31–35], most of which were not guided by a health theory. The information–motivation–behavioural skills model consists of three constructs, and was first proposed to help explain HIV preventive behaviours among students [36]. Its first construct, information, refers to the requisite knowledge about the particular health behaviour [37]; motivation is the second construct, of which attitude is a component; while the third construct, behavioural skills, comprises of both the actual and perceived abilities to carry out the behaviour change [36]. None of the previous health education interventions for malaria among pregnant women had targeted these three constructs as its outcome variable. This model, which suggests that information and motivation work through a mediator (behavioural skills), to cause a behaviour change has also been validated with diabetes self-care [38] and curb-cycle behaviour [39]. It was therefore hypothesized that giving health education on malaria to pregnant women using a module developed based on the IMB model is likely to improve their knowledge, motivation and behavioural skills towards those preventive measures. The aim of this study was as such, to develop, implement, and assess the effects of a health educational intervention based on the IMB model on knowledge, motivation, and behavioural skills scores among pregnant women in a hospital in north-eastern Nigeria.

## Methods

### Study location

The study was conducted in Maiduguri, the Borno state capital of north-eastern Nigeria. Borno state lies between latitudes 10° 30′ and 13° 50′ north and longitudes 11.00° and 13° 45′ east, with a total land area of 69,435 km^2^ [40]. Its population is reported to be 540,016 consisting of 282,409 males and 257,607 females [41]. The literacy rate among adult females in Maiduguri was 30.2% for English language, and 33.1% for any language [42]. The study location was the antenatal care clinic of the State Specialist Hospital Maiduguri, which is the biggest government secondary-level health centre in Borno state.

### Study design and selection of participant

The study was a randomized controlled parallel-group study conducted from 30 January 2017 to 14 June 2017. Participants were drawn from hospital’s antenatal care attendees. To ensure a uniform starting point, only those who were coming for their first antenatal care visit in their index pregnancies were included in the study. In addition, to maximize retention and allow for completion of the study before delivery, those not resident in Maiduguri as well as those with over 5 months period of amenorrhoea were excluded from the study. Those who do not speak Hausa language were also excluded, as the intervention was in Hausa language. Participants were selected over a period of 8 weeks, from 30 January, 2017 to 20 March, 2017 from the booking clinic sessions which are held on Mondays, in the hospital. The minimum required sample size for the study was calculated using the sample size formula for randomized controlled trials with continuous outcomes [43] for which the total knowledge scores from a previous intervention study were substituted [44]. This gave a total sample size of 296 for which an additional 30% was added for anticipated attrition to give a final sample size of 384 participants. After the selection of participants, baseline data was collected by trained enumerators using a structured questionnaire (Additional file 1), before randomly allocating them to either the intervention or control group.

### Randomization

#### Sequence generation and allocation concealment

The randomization sequence was generated by a trained staff of the hospital’s Medical Records Department, who was not part of any of the other research processes. The sequences consisted of permuted blocks of size four, each containing two interventions and two controls. The random function of Microsoft Excel was used to generate the random sequences of these permutations after which they were then placed in opaque envelopes and sealed.

#### Implementation

After the sequence generation, two other staff of the antenatal clinic who were also not involved in any other part of the study were responsible for the allocation. The first staff serially handed over the opaque envelopes to the respective participants without opening them, and then directed them to meet the second staff. The second staff then opened each envelope, gave the participant her study hand card, then documented her serial number and the group to which she belonged, and accordingly, informed her of the date to come her health education session.

#### Blinding

The study was double-blinded, as participants as well as the enumerators, were blinded to the groups they belonged, as it was not disclosed to them and it was not indicated on their study cards. The enumerators were also not involved in any other part of the study.

#### Intervention

The intervention group received a 4-h health education on malaria. The session was strictly guided by a module developed by the researchers based on the information–motivation–behavioural skills (IMB) model. The module had four sections, the first two of which covered aspects of malaria transmission; clinical features; complications of malaria during pregnancy, and preventive measures. These sections were named, ‘understanding malaria in pregnancy’ and ‘the main preventive measures for malaria in pregnancy’ respectively, and lasted for approximately 30 min each. The third section was named, ‘Insecticide treated net and Fansidar’, and lasted for about an hour and a half. Participants were taken through the details of how to use, and take care of their ITNs and how to take IPT, with regards to identifying the correct drug, timing, dosing, side-effects, and what to do in case they experienced one. The fourth section which lasted for about an hour and a half was named, was an interactive session named, ‘motivation for malaria prevention during pregnancy’. During this session, deterrent factors to the use of ITN and IPT, as identified from previous studies were highlighted, followed by brainstorming among the participants and the facilitator, on possible ways of overcoming those deterrent factors. The module was prepared and delivered in Hausa language, and it was pre-tested with a sample of 25 pregnant women who were not part of the study participants and appraised by a mid-wife, health educationist, and an Obstetrics and Gynecology specialist for necessary corrections and modifications.

The IMB model was chosen because it conceptualizes the psychological determinants of performing health behaviours [37]. It has three components which are: information about the health behaviour, motivation to carry out the behaviour, and the requisite skills for performing the behaviour [36]. A single session health education programme based on the IMB model was also effective in improving HIV preventive practices even 10 months post intervention [45]. In a systematic review of intervention studies based on the IMB model, ten out of the twelve included studies were effective in leading to positive behavioural changes, suggesting that the IMB provides a strong theoretical framework for behavioural interventions [46]. A theory such as the Protection Motivation Theory however, while laying much emphasis on motivation, fails to identify other environmental and cognitive factors that can affect attitude change [47].

#### Control group

The control group received health education on breastfeeding, which was designed to last for approximately the same duration as the health education on malaria. It was also delivered by the same facilitator.

#### Outcome variables and instrument

Follow-up data was collected from participants at 2-months (first follow-up) and at 4 months post-intervention (second follow-up) by the same enumerators, using the same questionnaire. The outcome variables in the study were total knowledge, motivation and behavioural skills scores. The questionnaire consisted of four sections, the first of which was for participants’ socio-demographic characteristics; the second was on knowledge of malaria with questions on malaria transmission, symptoms, complications, ITN and IPT. It had a total of 36 questions, with each question having three options: Yes, No, and I don’t know. A correct answer was scored one-point, while a wrong answer or ‘I don’t know’ was scored zero. The sum was calculated to obtain the total knowledge scores for that participant. The third section was on motivation, in which personal motivation was assessed by asking participants, how good or bad, and how pleasant or unpleasant sleeping under an ITN and taking IPT was, for them. These comprised of a total of six items, assessed on a Likert scale of 1–5. Social motivation was assessed with four items assessed on a Likert scale of 1–6. Participants were asked how true or otherwise it was that their significant others would support their use of ITN or IPT. The fourth section assessed behavioural skills with a total of seven items, the first three of which asked of the level of ease or otherwise of carrying the preventive measures, while the next four asked of how effectively or otherwise the participant could use and care for their ITN. For motivation and behavioural skills, the sum of all the points were calculated to obtain the total motivation and total behavioural skills scores.

### Statistical analyses

The data obtained (Additional file 2) was analysed using IBM SPSS version 22. Each of these total scores were converted to percentages of the total obtainable scores for that construct, for further statistical analyses. Normality tests performed on the total knowledge, motivation and behavioural skills scores, using the test of Kurtosis and histogram plots showed there was no substantial non-normality, and as such the data could be handled as normal data. Chi squared test was performed to compare the baseline characteristics as well as responses to other items of the questionnaire between the intervention and control group. Repeated measures analysis of variance (ANOVA) was performed to determine the main effect of group, time, and group-time interaction, on the respective scores. For both the mixed-design and one-way repeated measures ANOVA for knowledge, motivation and behavioural skills scores, the assumptions of sphericity were violated (Mauchly’s test *p*-value < 0.001) and the Epsilon values were all less than 0.75, and as such, the Greenhouse–Geisser corrected estimates were used in the interpretation of those results. Missing values were then replaced using the multiple imputations method and then the generalized linear mixed models (GLMM) analysis was performed to determine the effect size of the intervention. For sensitivity analysis, the GLMM analysis was repeated without missing value replacement. Permission to conduct the study as well as ethical approval, was obtained from the Ethical Committee of the State Specialist Hospital (SSH/GEN/64/Vol.1) and the Universiti Putra Malaysia [UPM/TNCPI/RMC/1.4.18.2 (JKEUPM)]. The trial was also registered with the Pan African Clinical Trial Registry (PACTR201610001823405). Informed consent was also obtained from the participants, after they had been taken through the respondent information sheet which was in Hausa language; and this was documented in the consent form by the enumerators.

## Results

### Response rate

**Fig. 1:**
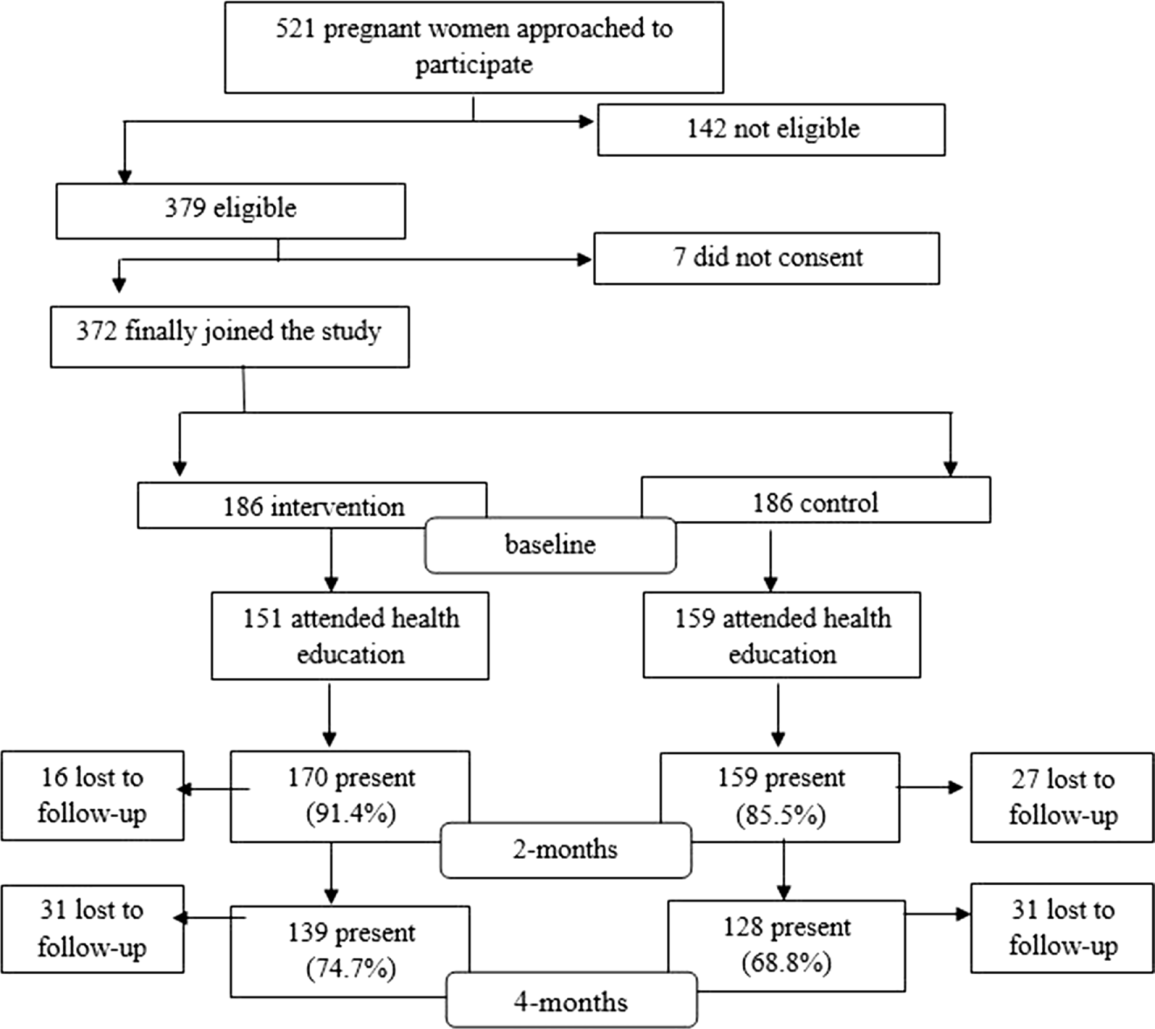
Flow chart of recruitment of respondents

The flow chart for participant recruitment is presented in Fig. 1. Three hundred and seventy-two pregnant women were finally selected to participate in the study, with 186 in the intervention group and 186 in the control group. As presented in the chart, 81.2% and 85.5% of participants from the intervention and control groups, respectively, had attended their respective health education sessions. At the end of the study, the total drop out from the intervention and control groups were 25.3% and 31.2% respectively.

### Baseline socio-demographic characteristics of participants

The ages of the respondents ranged from 15 to 45 years, with mean (SD) age of 26.5 (5.9) years. There was no divorced or single woman among the respondents, though two of them were widowed (0.5%). Most of the respondents (77.2%) were at the time of the research, married in monogamous settings, while 22.4% were in polygamous marriages. About a third (26.3%) of all the respondents were internally displaced persons (IDP) from other local government areas of Borno State, Nigeria. Over a half (58.9%) of them had some form of education, from primary to tertiary, but less than a half (44%) were employed. As shown in Table 1, with the exception of place of residence, for which there were more IDPs in the control group, both groups were comparable on their socio-demographic characteristics.

**Table 1:** Baseline socio-demographic characteristics of the participants

Socio-demographic factor	Groups		*χ* ^*2*^	*df*	*p*
	Intervention Freq. (%) (n = 186)	Control Freq. (%) (n = 186)			
Age (years)			− 1.399	370	0.163^a^
Mean (SD)	26.1 (5.8)	26.9 (5.9)			
(Range)	(15–40)	(17–45)			
Ethnicity			4.054	6	0.669
Kanuri	73 (37.7)	63 (33.9)			
Hausa	30 (16.1)	28 (15.1)			
Babur	13 (7.0)	18 (9.7)			
Shuwa	6 (3.2)	12 (6.4)			
Marghi	14 (7.5)	12 (6.4)			
Fulani	21 (11.3)	17 (9.1)			
Others	32 (17.2)	36 (19.4)			
Family type					
Monogamous	143 (76.9)	144 (77.4)	2.016	2	0.365
Polygamous	41 (22.0)	42 (22.6)			
Widowed	2 (1.1)	0 (0.0)			
Educational status			3.365	3	0.339
None	82 (44.1)	71 (38.2)			
Primary	32 (17.2)	32 (17.2)			
Secondary	54 (29.0)	54 (29.0)			
Tertiary	18 (9.7)	29 (15.6)			
Employment status			2.380	4	0.666
None	104 (55.9)	101 (54.3)			
Self-employed	56 (30.1)	65 (34.9)			
Government	9 (4.9)	9 (4.9)			
Private	14 (7.5)	10 (5.4)			
Student	3 (1.6)	1 (0.5)			
Income			0.768	2	0.681
None	106 (57.0)	102 (54.8)			
< 18,000	65 (34.9)	72 (38.7)			
≥ 18,000	15 (8.1)	12 (6.5)			
Type of residence			5.541	1	0.019*
Permanent resident	127 (68.3)	147 (79.0)			
IDP	59 (31.7)	39 (21.0)			

* Significant *p *< 0.05

^a^*p* for t-test

### Knowledge, motivation and behavioural skills at baseline, 2-months and 4-months post-intervention

At baseline, almost all the respondents (94.1%) stated that malaria could be transmitted through mosquito bites. Higher proportions of participants in the control group gave correct answers to each of the questions on malaria cause and transmission, though this difference was only significant for three out of the eight questions. At their first follow-up visit however, the proportions of correct answers were significantly higher among the intervention group, all of which were statistically significant, except the question on whether malaria transmitting mosquitoes can bite during the day. Over three-fourths of participants in both groups correctly identified the malaria symptoms mentioned in the questionnaire, with no significant difference between the groups at baseline. However, the intervention group showed a higher percentage of persons who gave correct answers at the time of first and second follow-up visits.

At baseline, even though there were significantly more in the control group who correctly identified anaemia and low birth weight as complications of malaria during pregnancy, this was reversed, at the time of the first and second follow-up visits. For knowledge of insecticidal nets, there was no significant difference between the groups at baseline, except for the items under net care, where more participants from the control group knew that ITNs should not be dried under the sun. The intervention group however exhibited better levels of knowledge about ITNs at the times of their subsequent visits. At baseline, significantly more participants among the control group correctly identified that not two tablets are given for IPTp and that the medicines are not harmful to pregnancy. However, at the subsequent visits, the correct responses given to these questions were significantly higher among the intervention group.

For the items of personal motivation which assessed participants’ perception of the level of goodness or otherwise, and perception of level of pleasantness or otherwise of sleeping under an ITN and taking IPTp, there were no differences between the groups at baseline. At the subsequent follow-up visits however, all the items were significantly better for the intervention group. There remained no difference between the groups at all the three time points, in terms of social motivation which assessed the support they were likely to get from their significant others in complying with ITN and IPTp. The degree of ease or otherwise of sleeping under an ITN and taking all the IPTp medicines was not different for both groups at baseline and 2-months post-intervention, but was better among the intervention group at 4-months post-intervention. Their level of effectiveness for ITN use and ITN care was the same, but subsequently remained better for the intervention group.

### Baseline comparison of mean knowledge, motivation and behavioural skills scores between groups

At baseline, the participants’ knowledge scores ranged from 4.4 to 93.3% with mean (SD) percentage score of 60.0 (15.8) percent. Their total motivation scores ranged from 44.4 to 100%, with mean (SD) percentage score of 81.6 (10.8) percent, while total behavioural skills scores ranged from 42.9 to 100%, with mean (SD) percentage score of 83.7 (12.7) percent. As presented in Table 2, there were no significant differences between the mean knowledge, motivation and behavioural skills scores of the groups at baseline.

**Table 2:** Baseline comparison of the mean total knowledge, motivation and behavioural skills scores of the intervention and control groups

Scores	Mean		*df*	*t*-value	Mean difference (95% CI)	*p*-value
	Intervention n = 186	Control n = 186				
Knowledge	59.02 (14.34)	61.05 (17.15)	358.77	− 1.24	− 2.03 (− 5.25, 1.19)	0.216
Motivation	81.55 (10.55)	81.55 (11.02)	369.30	< 0.001	0.00 (− 2.20, 2.20)	1.000
Behavioural skills	83.39 (11.94)	83.98 (13.39)	370.98	− 0.45	− 0.60 (− 3.18, 1.99)	0.651

### Follow-up change in total knowledge, motivation, and behavioural skills scores for the two groups

Results of independent t-test to determine group simple effect on total knowledge, motivation, and behavioural skills scores are presented in Table 3. The mean knowledge, motivation, and behavioural skills scores of the intervention group were higher than that of the control group at 2-months, and 4-months post-intervention. Also as shown in Table 4, the difference in mean knowledge, motivation, and behavioural skills scores over the repeated times were significant for both the intervention as well as control group.

**Table 3:** Comparison of mean knowledge, motivation, and behavioural skills scores of intervention and control groups at 2 months, and 4 months post-intervention

Variable	Mean		*df*	*t*-value	Mean difference (95% CI)	*p*-value
Knowledge scores						
2 months post-intervention	Intervention n = 170	Control n = 159				
	85.57 (13.13)	68.79 (17.88)	288.90	9.65	16.78 (13.35–20.20)	< 0.001
4 months post-intervention	Intervention n = 139	Control n = 128				
	92.00 (10.40)	77.47 (20.33)	185.69	7.26	14.54 (10.59–18.49)	< 0.001
Motivation scores						
2 months post-intervention	Intervention n = 170	Control n = 159				
	86.21 (7.96)	82.77 (10.26)	297.88	3.37	3.43 (1.43–5.44)	0.001
4 months post-intervention	Intervention n = 139	Control n = 128				
	84.73 (7.46)	82.41 (8.90)	248.73	2.30	2.32 (0.34–4.31)	0.022
Behavioural skills scores						
2 months post-intervention	Intervention n = 170	Control n = 159				
	90.17 (9.66)	83.85 (11.58)	308.49	5.35	6.31 (4.00–8.64)	< 0.001
4 months post-intervention	Intervention n = 139	Control n = 128				
	86.20 (10.93)	78.46 (6.68)	231.25	7.05	7.68 (5.58–9.90)	< 0.001

**Table 4:** Change in mean knowledge, motivation, and behavioural skills scores from baseline to 4-months post-intervention for both groups

Source variable	F	*df*	*p*-value	Partial Eta η^2^
Knowledge scores				
Intervention	82,719.415	1.667	< 0.001	0.693
Control	15,947.520	1.681	< 0.001	0.291
Motivation scores				
Intervention	12.573	1.872	< 0.001	0.086
Control	0.529	1.903	0.581	0.005
Behavioural skills scores				
Intervention	13.962	1.656	< 0.001	0.094
Control	12.488	1.857	< 0.001	0.096

**Fig. 2:**
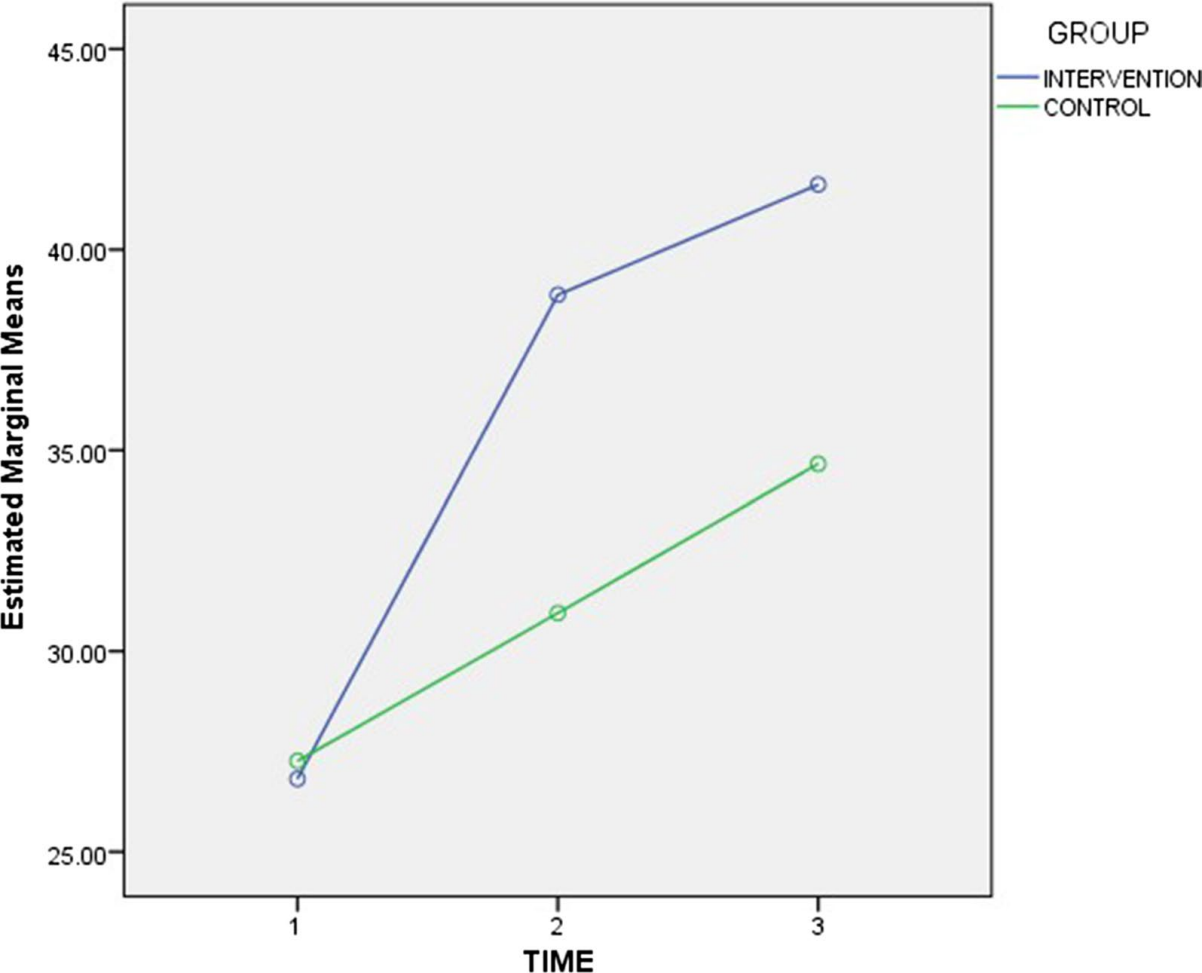
Trend of mean knowledge scores for intervention and control groups. The mean knowledge scores showed an increasing trend from baseline to the time of second follow-up for both groups

**Fig. 3:**
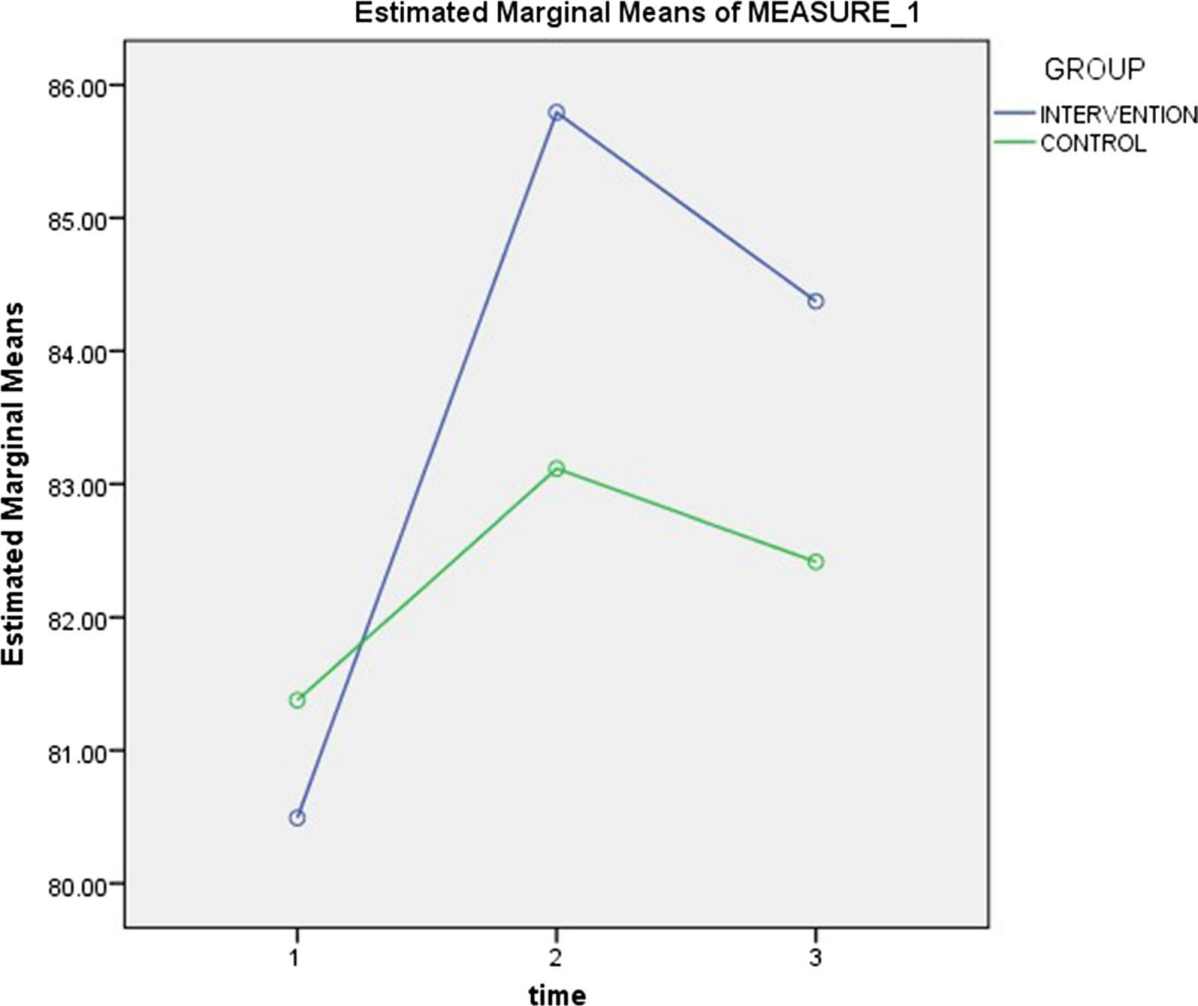
Trend of mean motivation scores for intervention and control groups. While there was a continuous rise in motivation scores for the control group, there was an initial rise followed by a drop, for the intervention group

**Fig. 4:**
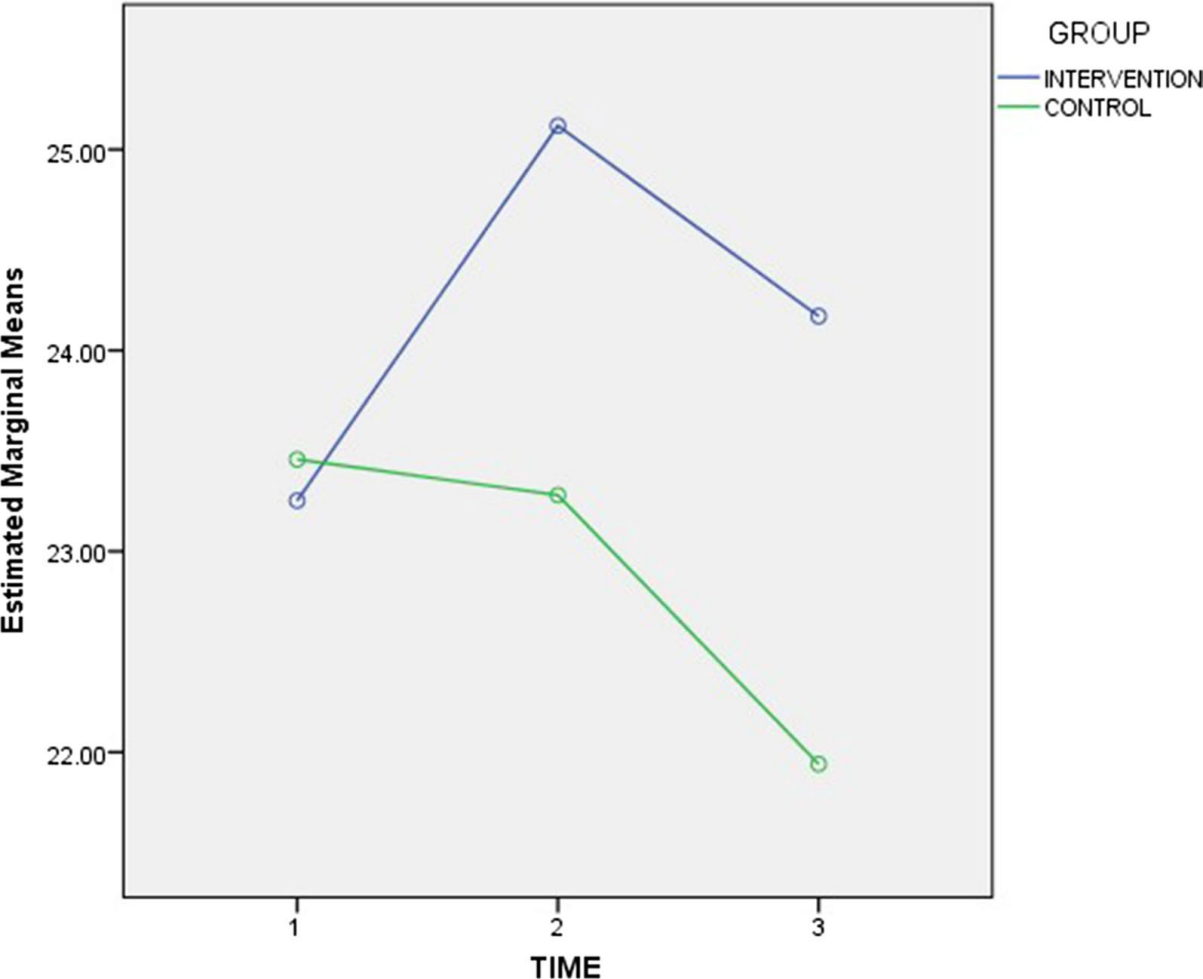
Trend of mean behavioural skills scores for intervention and control groups. The behavioural skills scores, shows that while there was an initial rise, followed by a fall, for the intervention group, there was a continuous drop from baseline to the time of the second follow-up visit, for the control group

The mean knowledge scores showed an increasing trend from baseline to the time of second follow-up for both groups, as shown in Fig. 2 (see Table 5). As illustrated in Fig. 3 (see Table 6), while there was a continuous rise in motivation scores for the control group, there was an initial rise followed by a drop, for the intervention group. However, for behavioural skills scores, Fig. 4 shows that while there was an initial rise, followed by a fall, for the intervention group, there was a continuous drop from baseline to the time of the second follow-up visit, for the control group (see Table 7).

**Table 5:** Effect of group with regards to time on the mean knowledge scores

Source variable: knowledge scores	F	*df*	*p*-value	Partial Eta η^2^
Group	44.332	1, 249	< 0.001	0.151
Residence	1.196	1, 249	0.275	0.005
Group*residence	0.653	1, 249	0.420	0.003
Time	184.182	1.712, 426.204	< 0.001	0.425
Time*group	36.752	1.712, 426.204	< 0.001	0.129

Results adjusted for type of residence

**Table 6:** Effect of group with regard to time on the mean motivation scores

Source variable self-efficacy scores	F	*df*	*p*-value	Partial Eta η^2^
Group	1.636	1, 249	0.202	0.007
Residence	2.808	1, 249	0.095	0.011
Time	8.659	2, 498	< 0.001	0.034
Time*group	2.356	2, 498	0.096	0.009

Results adjusted for type of residence

**Table 7:** Effect of group with regard to time on the mean behavioural skills scores

Source variable: behavioural skills scores	F	*df*	*p*-value	Partial Eta η^2^
Group	25.322	1, 249	< 0.001	0.092
Residence	1.378	1, 249	0.242	0.006
Time	10.039	1.784, 444.203	< 0.001	0.039
Time*group	9.733	1.784, 444.203	< 0.001	0.038

Results adjusted for type of residence

### Main effects of the intervention

For the generalized linear mixed models (GLMM) analysis, the participants’ baseline characteristics were controlled for, using the combination of variables which gave the best model, evidenced by lowest Akaike corrected information criterion (ACIC) and Bayesian information criterion (BIC). Table 8 shows that controlling for these factors, group and time had significant effects on total knowledge, motivation, and behavioural skills scores. The significant interaction between group and time also shows that the changes in these scores, were different between the groups over time.

**Table 8:** Fixed effects of group, time, and group-time interaction on total knowledge, motivation and behavioural skills scores

Source	F	df1	df2	Sig.
Knowledge				
Group	85.85	1	1063	< 0.001
Time	200.87	2	1063	< 0.001
Group*time	31.80	2	1063	< 0.001
Motivation				
Group	52.07	1	1062	< 0.001
Time	1119.76	2	1062	< 0.001
Group*time	23.44	2	1062	< 0.001
Behavioural skills				
Group	48.38	1	1062	< 0.001
Time	29.00	2	1062	< 0.001
Group*time	13.66	2	1062	< 0.001

Missing data replaced

### Magnitude of the intervention effect

Table 9 presents the fixed coefficients of the outcome variables studied. The results show that controlling for all other variables, a person in the intervention group was expected to have total knowledge, motivation, and behavioural skills scores of 12.75%, 8.55% and 6.35% respectively, above one in the control group.

**Table 9:** Fixed coefficients of outcome variables

Variable	Coefficient	Std. error	*t*	Sig.	95% CI	
					Lower	Upper
Knowledge						
Intervention	12.75	1.659	7.686	< 0.001	9.50	16.01
Control	1					
Motivation						
Intervention	8.55	0.921	9.286	< 0.001	6.74	10.36
Control	1					
Behavioural skills						
Intervention	6.35	0.989	6.419	< 0.001	4.41	8.29
Control	1					

Missing data replaced

### Sensitivity analysis

As presented in Table 10, the effects of the group on the outcome variables remained significant even after the GLMM analysis was conducted without replacing missing values. A comparison of the fixed coefficients for group, with, and without replacement of missing values is presented in Table 11. A decrease in the effect of the intervention on motivation as a result of drop out was seen only for motivation scores.

**Table 10:** Fixed effects of group, time, and group-time interaction on total knowledge, motivation and behavioural skills scores

Source	F	df1	df2	Sig.
Knowledge				
Group	144.92	1	914	< 0.001
Time	296.22	2	914	< 0.001
Group*time	50.27	2	914	< 0.001
Motivation				
Group	10.64	1	914	0.001
Time	11.87	2	914	< 0.001
Group*time	4.38	2	914	0.013
Behavioural skills				
Group	60.45	1	914	< 0.001
Time	19.93	2	914	< 0.001
Group*time	19.84	2	914	< 0.001

Missing data not replaced

**Table 11:** Comparison of fixed coefficients for group, with and without imputed missing values

Variable	Missing values not imputed		Missing values imputed		Coefficient difference	Percentage coefficient difference
	Coefficient	Sig.	Coefficient	Sig.		
Knowledge						
Intervention	15.66	< 0.001	12.75	< 0.001	2.91	22.82
Control	1		1			
Motivation						
Intervention	2.47	0.010	8.55	< 0.001	− 6.28	− 73.45
Control	1		1			
Behavioural skills						
Intervention	8.252	< 0.001	6.35	< 0.001	1.902	29.95
Control	1		1			

## Discussion

That almost all the participants in the study were aware of mosquito bites as a means through which malaria infection could be transmitted is not surprising, considering the Hausa name for malaria being ‘*zazzabin cizon sauro*’, a term, literally translated to mean, ‘fever caused by mosquito bite’. At the first follow-up visit, all respondents in the intervention group correctly identified mosquito bites as a means of malaria transmission, while four respondents in the control group answered the question wrongly; a finding similar to that of a previous study among refugee mothers in Central America [32]. The intervention was also impactful in raising their knowledge about ITNs at the times similar to findings from a previous study [35].

There was significant increase in the total knowledge scores for both groups, which is not unexpected, since the routine health talks given during the ante-natal care visits, also include discussions on malaria in pregnancy. Attending antenatal care has been also been reported to predict having higher accurate knowledge of malaria [48]. Previous studies have shown that even health education interventions on malaria which were not guided by any health theory, were still effective in leading to higher malaria knowledge [31, 35]. In Bangladesh, a community-wide distribution of insecticidal nets alongside health education by the distributors, was effective in leading to increased knowledge of malaria and insecticidal nets [33]. In contrast to a prior study which showed no difference between the effects of a plain health education session and one based on the IMB model [45], this study had demonstrated the superiority of the IMB-based health education over a plain one in improving knowledge levels. The decreased effect size after replacing missing values is indicative of a likely lesser outcome among those who dropped out, similar to findings from a previous study where the odds of having higher knowledge of malaria transmission and prevention were higher among those who had completed the intervention programme, compared to those who had not [35].

Level of motivation showed a quite different trend from that of knowledge, with different patterns for both groups. The scores for the intervention group however remained higher all through after the baseline, indicating the effectiveness of the intervention. This was probably because behavioural belief, which is an important sub-item of the motivation construct [49], had been extensively addressed in the intervention module. Considering the nature of the intervention, improvements should only be expected in personal motivation and not social motivation, which is dependent on others, not involved in the study [37]. This could explain the relatively lesser effect of the intervention on motivation scores, compared to knowledge scores. It seems a special intervention, especially one guided by a health theory, is requisite for achieving increases in motivation. This could be inferred from this study as well as a previous study, where a plain health education, led to no increase in attitude towards malaria [31], but another, guided by a health theory, led to an increase in motivation [34]. Similarly, an intense 5-h health educational intervention with lectures and practical exercises did not lead to any change in attitudes towards malaria prevention [35]. The results for motivation scores also point to the robustness of the GLMM analysis over the mixed method repeated measures ANOVA.

The trend for behavioural skills scores indicates that the routine health education given to the antenatal attendees was not impactful in increasing their level of behavioural skills, since only a continuous decrease in the mean scores was seen throughout the study period for the control group. Self-efficacy, an important component of behavioural skills, was reported to increase among the intervention group, following a malaria health educational intervention based on the protection motivation theory [34], indicating that guiding a health education intervention with a valid health theory may be necessary to improve behavioural skills.

The similarities in the baseline characteristics of the intervention and control groups suggest that the randomization was adequate. The Nigerian Demographic and Health Survey of 2013 [18] sampled respondents between the ages of 15–49 years, which was similar to the age range in this study. Respondents from both studies also had similar average ages at first marriage (17 versus 17.3), and proportion of those married in monogamous settings (77.2% versus 73.6%). This comparability of participants’ characteristics with those from the national survey, suggests the possibility of a wider generalization of its results.

Some strengths of the study included the randomization and blinding of participants. Also, in contrasts to many health education modules in Nigeria which are prepared in English language, with translation and interpretation left solely at the discretion of the facilitator, the module for this study was developed ab initio in the target language, which would have ensured greater uniformity in its delivery. The placebo intervention given to the control group on a relevant topic like breast feeding, is likely to have minimized resentful demoralization among them. Having had all sessions for both groups delivered by a single facilitator is likely to have eliminated or minimized experimenter effects. With regards to data analysis, the robust method used (generalized linear mixed models analysis) which handles both clustering effect as well as random effects, while also controlling for multiple confounding factors, was likely to allow for visualization of the pure effects of the intervention. Also, the intention to treat analysis ensured that the randomization was preserved. The sensitivity analysis allowed for estimating the impact of attrition on the results obtained, giving an idea of their robustness.

Among the limitations of this study were the inability to maintain an absolutely controlled environment, as the results from the control group suggest that other external factors had played some roles in influencing the outcome variables. The possibility of contamination from information sharing which could occur at home or other meeting places cannot also be excluded. At 4 months post-intervention, the number of respondents still retained in the study was a little short of the minimum calculated sample size (267 against 296), which could have dampened the effect of the intervention, as the chances of a type II error were higher. Attrition is also likely to have affected the accuracy of the results, as the multiple imputations technique was used to replace missing data points of participants who had dropped out of the study. The extrapolated data points were unlikely to have been a hundred percent accurate since they were not actually measured, but estimated. A drawback of the IMB model is its failure to account for environmental and cultural factors, which are important in predicting and explaining behaviours [37].

## Conclusion

From the findings of higher knowledge, motivation and behavioural skills scores among the intervention group at the time of the first and second follow-up visits, it can be concluded that the intervention was effective in improving the study outcomes. There is however room for improvement, considering the magnitude of the respective effect sizes. It is, therefore, recommended for the study module to be adopted and incorporated into the routine ante-natal care programs. Also considering the relatively shorter duration of sustainability of the effects of the intervention on motivation and behavioural skills level, it is recommended that at least an additional session be delivered say, 2–3 months after the first, to serve as a re-enforcement. Qualitative studies need to be conducted to explore the extraneous factors which had led to positive increases in the study outcomes among the control group. This would open doors to the development of more effective interventions in the future. Since the module was developed within the Nigerian context, there exists the need to have the module translated into other Nigerian languages and evaluated for its effectiveness.

## Additional files

**Additional file 1.** Study questionnaire.

**Additional file 2.** Raw data of the study.
