# Supplementary material for: Effects of a health educational intervention on malaria knowledge, motivation, and behavioural skills: a randomized controlled trial
Source: Malar J. 2019 Feb 20;18:41. doi: 10.1186/s12936-019-2676-3 (PMC6381678; doi:10.1186/s12936-019-2676-3)
Supplement: Supplementary file 1 — Additional file 1. Study questionnaire. [file 12936_2019_2676_MOESM1_ESM.docx]

**QUESTIONNAIRE**

**(ENGLISH)**

**Instructions:** Please answer ALL questions and please TICK (√) only one answer for each statement, in the appropriate box.

**Reminder:** Please try to answer all questions honestly as this is not an examination or a test.

**SECTION A**

**Instructions:** Please tick (√) the most appropriate option or fill in your response.

1. Date of data collection (dd/mm/yyyy) ……../………../…………..

2. Age: …….... years

3. Ethnicity

- Kanuri
- Hausa
- Babur
- Shuwa
- Marghi
- Fulani
- Others (Specify) ……………….

4. Marital status

- Single
- Married
- Widowed
- Divorcee

5. Family type

- Monogamy
- Polygamy

6. Residence in Maiduguri

- Permanent Resident
- Internally displaced person

7. Educational status

- No formal educational
- Primary
- Secondary
- Tertiary

8. Occupation

- None/housewife
- Self-employed
- Government employed
- Private employment
- Student

9. Monthly income in naira ……………

**SECTION B**

**Instructions:** Please tick (√) the most appropriate option based on the options provided which are: **YES**, **NO** or **I DON’T KNOW** for each of the statements below.

| **S/NO** | **Questions** | **Response** | | |
| --- | --- | --- | --- | --- |
|  |  | **YES** | **NO** | **I DON’T KNOW** |
| 10. | **How is malaria transmitted?** | | | |
|  | Mosquito bite |  |  |  |
|  | Being soaked in rain |  |  |  |
|  | Changing weather |  |  |  |
|  | Eating certain foods |  |  |  |
|  | Working hard under the sun |  |  |  |
|  | Others………. |  |  |  |
| 11. | **What are the symptoms of malaria?** | | | |
|  | Hotness of the body |  |  |  |
|  | Shivering |  |  |  |
|  | Headache |  |  |  |
|  | Joint pains |  |  |  |
|  | Loss of appetite |  |  |  |
|  | Feeling of unwell |  |  |  |
|  | Bitter taste |  |  |  |
|  | Nausea |  |  |  |
|  | Vomiting |  |  |  |
|  | Could look healthy |  |  |  |
| 12. | Can malaria mosquitoes also bite during the day? |  |  |  |
| 13. | Does pregnancy increase the chances of contracting malaria? |  |  |  |
| 14. | Does malaria have harmful effects on the pregnant woman? |  |  |  |
| 15. | Does malaria have harmful effects on the foetus? |  |  |  |
| 16. | **What harm can malaria cause during pregnancy?** | | | |
|  | It can cause the pregnant woman to have insufficient blood |  |  |  |
|  | Miscarriage |  |  |  |
|  | Pre-term delivery |  |  |  |
|  | Can cause the baby to have a low birth weight |  |  |  |
|  | Death of mother |  |  |  |
|  | Death of foetus |  |  |  |
| 17. | Are you aware of long lasting insecticidal bed nets? |  |  |  |
| 18. | **What are insecticidal nets used for?** | | | |
|  | To keep mosquitoes away |  |  |  |
|  | To keep rats away |  |  |  |
| 19. | Insecticidal nets are more effective compared to plain nets |  |  |  |
| 20. | The chemicals on insecticidal bed nets can be dangerous to one who sleeps under it |  |  |  |
| 21. | **After how long should insecticidal nets be washed?** | | | |
|  | After 1 month |  |  |  |
|  | After 3-4 months |  |  |  |
|  | After 6 months |  |  |  |
| 22. | **With what should insecticidal nets be washed?** | | | |
|  | Water and ordinary soap only |  |  |  |
|  | Water and detergent |  |  |  |
| 23. | **Where should insecticidal nets be dried, after washing?** | | | |
|  | Under the shade |  |  |  |
|  | Under the sun |  |  |  |
| 24. | Are you aware of the treatment given during pregnancy for protection against malaria? |  |  |  |
| 25. | **Which medicine is given for protection against malaria during pregnancy?** | | | |
|  | Chloroquine |  |  |  |
|  | Fansidar |  |  |  |
| 26. | **How many tablets of the medicine for protection against malaria during pregnancy should be given at each time?** | | | |
|  | 2 tablets |  |  |  |
|  | 3 tablets |  |  |  |
|  | 1. tablets |  |  |  |
| 27. | The medicine given to pregnant women for protection against malaria during pregnancy can be harmful to the pregnancy |  |  |  |
| 28. | Can the medicine given for protection against malaria in pregnancy be taken on an empty stomach? |  |  |  |

**SECTION C**

**Instructions:** Please TICK (√) only one answer for each statement. Each statement is answered based on a 5-point scale which ranges from 1 = very bad to 5 = very good; or from 1 = very unpleasant to 5 = very pleasant.

| a | **Please tell us how good or bad the following are for your health** | **very bad** | **somewhat bad** | **neither bad nor good** | **somewhat good** | **very good** |
| --- | --- | --- | --- | --- | --- | --- |
|  | For the remaining duration of your pregnancy, how good or bad would it be for your health…. |  |  |  |  |  |
| 29. | To sleep under an insecticidal net? |  |  |  |  |  |
| 30. | To sleep more frequently under an insecticidal net? |  |  |  |  |  |
| 31. | To take all the medicines given to you for preventive treatment of malaria in pregnancy? |  |  |  |  |  |
| 32. | Take all the medicines given to you for preventive treatment of malaria in pregnancy even when you don’t feel sick |  |  |  |  |  |
| **b** | **Please tell us how pleasant or unpleasant it would be for you to do the behaviour** | **very unpleasant** | **somewhat pleasant** | **neither unpleasant nor pleasant** | **somewhat pleasant** | **very pleasant** |
|  | For the remaining duration of your pregnancy, how pleasant or unpleasant would it be for you…. |  |  |  |  |  |
| 33. | To sleep under an insecticidal net? |  |  |  |  |  |
| 34. | To sleep more frequently under an insecticidal net? |  |  |  |  |  |

**Instructions:** Please TICK (√) only one answer for each statement. Each statement is answered based on a 6-point scale which is:

1: Very untrue

2: Mostly untrue

3: Untrue

4: True

5: Mostly true

6: Very true

|  | **Please tell us how true or untrue it is for you** | **very untrue** | **mostly untrue** | **untrue** | **true** | **mostly true** | **very true** |
| --- | --- | --- | --- | --- | --- | --- | --- |
|  | Most people who are important to you think you should… |  |  |  |  |  |  |
| 35. | Sleep under an insecticidal net |  |  |  |  |  |  |
| 36. | Sleep more frequently under an insecticidal net |  |  |  |  |  |  |
| 37. | Take all the medicines given to you for preventive treatment of malaria in pregnancy |  |  |  |  |  |  |
| 38. | Take all the medicines given to you for preventive treatment of malaria in pregnancy even when you don’t feel sick |  |  |  |  |  |  |

**SECTION D:**

**Instructions:** This section asks about level of difficulty/ease and effectiveness/ineffectiveness in performing certain tasks.

Please TICK (√) only one answer for each statement. Each statement is answered according to a four point scale which is:

1: Very hard

2: Hard

3: Easy

4: Very easy

| **a** | **Right now, how easy or hard would it be for you to…** | **Very hard**  **1** | **Hard**  **2** | **Easy**  **3** | **Very easy**  **4** |
| --- | --- | --- | --- | --- | --- |
| 39. | Sleep under an insecticidal bed net every night? |  |  |  |  |
| 40. | Take all the medicines given to you for prevention against malaria during pregnancy? |  |  |  |  |
| 41. | Take all the medicines given to you for prevention against malaria during pregnancy even when you experience mild discomfort taking them? |  |  |  |  |
| **b** | **Right now, how effectively or ineffectively could you…** | **Very Ineffectively**  **1** | **Ineffectively**  **2** | **Effectively**  **3** | **Very Effectively**  **4** |
| 42. | Properly hang your insecticidal net? |  |  |  |  |
| 43. | Check for and repair holes and rifts in your insecticidal bed net? |  |  |  |  |
| 44. | Sleep more frequently under an insecticidal bed net? |  |  |  |  |
| 45. | Persuade others to support your sleeping under an insecticidal bed net? |  |  |  |  |

Thank you for your co-operation!

**JERIN TAMBAYOYI**

**(HAUSA)**

**Umarni:** Don Allah, a amsa dukkan tambayoyi, kuma a alamta (√) amsa guda daya kawai ga kowane kalamia ɗan akwatin da ya dace.

**Matashiya:** Don Allah, a ƙoƙarta a amsa dukkan tambayoyin tsakani da Allah domin wannan ba jarrabawa ba ce ko gwaji.

**SASHEN A**

**Umarni:** Don Allah a alamta (√) amsar da ta fi dacewa ko a cike amsar da kalamai.

1. Ranar tattara bayanai (rana/wata/shekara)………../………../…………………

2. Shekaru: ………….

3. Ƙabila

- Kanuri
- Hausa
- Babur
- Shuwa
- Marghi
- Fulani
- Sauran (a bayyana) ……………….

4. Matsayin aure

- Gwauro
- Ina da aure
- Dalilin Rasuwa
- Dalilin saki

5. Tsarin iyali

- Auren tilo
- Auren mata fiye da ɗaya

6. Zama a Maiduguri

- Mazaunin din-din-din
- Gudun hijira

7. Matsayin Ilimi

- Ban yi makaranta ba
- Na yi Firamare
- Na yi sakandare
- Na yi na gaba da sakandare

8. Matsayin aiki

- Babu/matar aure
- Ina aikin kai na
- Aikin gwamnati
- Aikin masana’anta
- Ɗalibta

9. Kuɗin da nake samu duk wata Naira….……………….

**SASHEN B**

**Umarni:** Don Allah a alamta haka ‘√’ ga amsar da ta dace daga cikin zaɓin da aka ba da, wato, kodai **EH, A’A**, ko **BAN SANI BA** ga kowane ɗaya daga kalaman da ke tafe a ƙasa

| **Lamba** | | **Tambayoyi** | **Amsoshi** | | |
| --- | --- | --- | --- | --- | --- |
|  |  | | **EH** | **A’A** | **BAN SANI BA** |
| 10. | **Ta yaya ake kamuwa da malariya?** | | | | |
|  | Cizon sauro | |  |  |  |
|  | Jiƙewa da ruwan sama | |  |  |  |
|  | Sauyin yanayi | |  |  |  |
|  | Cin wasu irin abinci | |  |  |  |
|  | Aikin wahala a rana | |  |  |  |
|  | Sauran……………. | |  |  |  |
| 11. | **Mene ne alamun cutar malariya?** | | | | |
|  | Zafin jiki | |  |  |  |
|  | Karkarwa | |  |  |  |
|  | Ciwon kai | |  |  |  |
|  | Ciwon gaɓoɓi | |  |  |  |
|  | Rashin son cin abinci | |  |  |  |
|  | Jin bani da lafiya | |  |  |  |
|  | Ɗacin baki | |  |  |  |
|  | Jin amai | |  |  |  |
|  | Yin amai | |  |  |  |
|  | Jin kamar lafiya ta ƙalau | |  |  |  |
| 12. | Shin sauron da ke yaɗa cutar malariya na iya cizo da rana? | |  |  |  |
| 13. | Shin goyon ciki na iya ƙara kawo kamuwa da cutar malariya? | |  |  |  |
| 14. | Shin cutar malariya na iya cutar da mai goyon ciki? | |  |  |  |
| 15. | Shin cutar malariya na iya cutar da ɗan tayin ciki? | |  |  |  |
| 16. | **Wace irin illa malariya kan iya jawowa lokacin goyon ciki?** | | | | |
|  | Tana iya sa mace mai ciki ta rasa isasshen jinni | |  |  |  |
|  | Yin ɓari | |  |  |  |
|  | Haihuwa ba lokacin da ya dace ba | |  |  |  |
|  | Haddasa haihuwar ɗa/’ya mai ƙarancin nauyi | |  |  |  |
|  | Mutuwar uwa | |  |  |  |
|  | Mutuwar ɗan tayi | |  |  |  |
| 17. | Kina da masaniyar gidan sauron da ke ɗauke da feshin maganin sauro? | |  |  |  |
| 18. | **Me ake yi da gidan sauron da ke ɗauke da feshin maganin sauro?** | | | | |
|  | Kawar da sauro | |  |  |  |
|  | Kawar da ɓeraye | |  |  |  |
| 19. | Gidan sauro mai feshin magani ya fi wanda ba feshin magani | |  |  |  |
| 20. | Feshin maganin gidan sauron kan iya zamowa haɗari gare ni muddin na kwanta a cikinsa | |  |  |  |
| 21. | **Bayan tsawon wane lokaci ya kamata a wanke gidan sauro mai feshin magani?** | | | | |
|  | Bayan wata 1 | |  |  |  |
|  | Bayan wata 3 | |  |  |  |
|  | Bayan wata 6 | |  |  |  |
| 22. | **Da me ya kamata a wanke gidan sauro mai feshin maganin sauro?** | | | | |
|  | Ruwa da sabulu | |  |  |  |
|  | Ruwa da omo | |  |  |  |
| 23. | **A ina ya kamata a shanya gidan sauro mai feshin magani?** | | | | |
|  | A inuwa | |  |  |  |
|  | A rana | |  |  |  |
| 24. | Kina da masaniya akan maganin da ake bayarwa na kariya lokacin goyon ciki? | |  |  |  |
| 25. | **Wane irin magani ake bayarwa don kariya daga cutar malariya lokacin goyon ciki?** | | | | |
|  | Chloroquine | |  |  |  |
|  | Fansidar | |  |  |  |
| 26. | **Nawa ne adadin kwayoyin maganin kariya daga cutar malariya da ake bayarwa kowane lokaci ga mai goyon ciki?** | | | | |
|  | Ƙwaya 2 | |  |  |  |
|  | Ƙwaya 3 | |  |  |  |
|  | Ƙwaya 4 | |  |  |  |
| 27. | Maganin da ake ba wa masu goyon ciki don kariya daga cutar malariya zai iya zama mai illa akan cikin da nake goyo | |  |  |  |
| 28. | Ana iya shan maganin kariya daga cutar malariya ba tare da an ci abinci ba? | |  |  |  |

**SASHEN C**

**Umarni:** Don Allah a alamta (√), amma amsa ɗaya ake bukata ga kowane kalami.

Kowane kalami za a amsa ne daidai da sikili mai hawa 5 wadda ya fara daga

1 = Ba kyau sosai, zuwa 5 = Da kyau sosai; DA KUMA

1 = Ba daɗi sosai, zuwa 5 = Da daɗi sosai.

| **a** | **Don Allah a gaya mana yaya kyaun ko rashin kyaun waɗannan game da lafiyarki.** | **Ba kyau sosai** | **Haka dai ba kyau** | **Ba kyau ba daɗi** | **Haka dai da kyau** | **Da kyau sosai** |
| --- | --- | --- | --- | --- | --- | --- |
| 29. | Rinƙa kwana a cikin gidan sauro mai feshin magani |  |  |  |  |  |
| 30. | Rinƙa kwana akai-akai fiye da da a cikin gidan sauro mai feshin magani |  |  |  |  |  |
| 31. | Rinƙa shan maganin kariya daga cutar malariya da aka ba ni yayin goyon ciki |  |  |  |  |  |
| 32. | Rinƙa shan dukkan magungunan kariya daga cutar malariya da aka bani ko da ina jin lafiyata ƙalau |  |  |  |  |  |
| **b** | **Don Allah a gaya mana yaya daɗi ko rashin daɗin waɗannan halayen a gareki.** | **Ba dadi sosai** | **Haka dai ba dadi** | **Ba kyau ba daɗi** | **Haka dai da dadi** | **Da dadi sosai** |
| 33. | Rinƙa kwana a cikin gidan sauro mai feshin magani |  |  |  |  |  |
| 34. | Rinƙa kwana akai-akai fiye da da a cikin gidan sauro mai feshin magani |  |  |  |  |  |

**Umarni:** Don Allah a alamta (√), amma amsa ɗaya ake bukata ga kowane kalami.

Kowane kalami za a amsa ne daidai da sikili mai ma’auni shida:

1: Ƙarya ne sosai

2: Akasari ƙarya ne

3: Ƙarya ne

4: Gaskiya ne

5: Akasari gaskiya ne

6: Gaskiya ne sosai

|  | **Don Allah a gaya mana yaya gaskiya ko rashin gaskiyar ta ke a gareki** | **Ƙarya ne sosai** | **Akasari ƙarya ne** | **Ƙarya ne** | **Gaskiya ne** | **Akasari gaskiya ne** | **Gaskiya ne sosai** |
| --- | --- | --- | --- | --- | --- | --- | --- |
|  | Mutanen da ke da muhimmanci a gare ki suna tsammanin yakamata ki… |  |  |  |  |  |  |
| 35. | Rinƙa kwana a cikin gidan sauro mai feshin magani |  |  |  |  |  |  |
| 36. | Rinƙa kwana akai-akai fiye da da a cikin gidan sauro mai feshin magani |  |  |  |  |  |  |
| 37. | Rinƙa shan maganin kariya daga cutar malariya da aka ba ki yayin goyon ciki |  |  |  |  |  |  |
| 38. | Rinƙa shan maganin kariya daga cutar malariya da aka baki ko da kina jin lafiyarki ƙalau |  |  |  |  |  |  |

**SASHEN D**

**Umarni:** A wannan sashe ana tambaya akan kimar wahala ko sauƙi da iyawa ko rashin iya gudanar da wasu ayyuka

Don Allah a alamta (√), amma amsa ɗaya ake bukata ga kowane kalami. Kowane kalami za a amsa ne daidai da sikili mai ma’auni huɗu.

| **a.** | **A halin yanzu yaya wahalar ko sauƙin yadda zaki iya….** | **Da wahala sosai** | **Da wahala** | **Da sauƙi** | **Da sauƙi sosai** |
| --- | --- | --- | --- | --- | --- |
|  |  | **1** | **2** | **3** | **4** |
| 39. | Kwana a cikin gidan sauro mai feshin magani kowace rana? |  |  |  |  |
| 40. | Shanye dukkan maganin kariya daga zazzaɓin cizon sauro lokacin goyon ciki? |  |  |  |  |
| 41. | Shanye dukkan maganin kariya daga zazzaɓin cizon sauro ko da kikan ji ba daɗi? |  |  |  |  |
| **b.** | **A halin yanzu yaya ƙwarewa ko rashin ƙwarewarki wajan….** | **Ban kware ba kwata-kwata** | **Ban kware ba** | **Na kware** | **Na kware sosai** |
| 42. | Rataya gidan sauro daidai? |  |  |  |  |
| 43. | Dubawa ko gyara huji da yagewar gidan sauro mai feshin magani? |  |  |  |  |
| 44. | Ƙara adadin kwanaki a sati da kike kwana a cikin gidan sauro a halin yanzu? |  |  |  |  |
| 45. | Shawo kan wasu don su goyi bayan kwana da kike a cikin gidan sauro? |  |  |  |  |

An Gode da haɗin kai da aka ba da!
